# Analysis of Caffeine, Chlorogenic Acid, Trigonelline, and Volatile Compounds in Cold Brew Coffee Using High-Performance Liquid Chromatography and Solid-Phase Microextraction—Gas Chromatography-Mass Spectrometry

**DOI:** 10.3390/foods9121746

**Published:** 2020-11-26

**Authors:** JeongAe Heo, Koushik Adhikari, Kap Seong Choi, Jeehyun Lee

**Affiliations:** 1 Department of Food Science and Nutrition & Kimchi Research Institute, Pusan National University, Busan 46241, Korea; jeongaeheo0510@gmail.com; 2 Department of Food Science and Technology, University of Georgia, Griffin, GA 30223, USA; koushik7@uga.edu; 3 School of Food Science, Sunchon National University, Suncheon 57922, Korea; chks@sunchon.ac.kr

**Keywords:** cold brew coffee, headspace solid-phase microextraction, gas chromatography-mass spectrometry, high-performance liquid chromatography

## Abstract

This study investigated the non-volatile and volatile compounds in samples of cold brew (CB) coffee, coffee from a coffee shop (CS), ready-to-drink (RTD) coffee, and brewed coffee from a coffee maker (CM). The volatile compounds were identified using headspace solid-phase microextraction with gas chromatography-mass spectrometry, and the samples were treated with high-performance liquid chromatography for the quantification of caffeine, chlorogenic acid, and trigonelline. The results indicate that RTD coffee had the lowest amounts of non-volatile compounds. A total of 36 volatile compounds were semi-quantified; the contents of most volatile compounds in CS and Folgers samples were higher than those in CB and CM samples. The contents of 25 volatile compounds in the CM sample were higher than those in the CB sample. The consumer and instrumental data show that the bitterness intensity was correlated with pyrazines, pyrroles, and guaiacols, whereas the coffeeID intensity was correlated with phenols. Semi-quantification and principal component analysis results show that the extraction method and temperature could influence the volatile compound profiles.

## 1. Introduction

Coffee is one of the most complex beverages because it contains many non-volatile and volatile compounds generated during the brewing process. Non-volatile components of coffee are composed of carbohydrates, proteins, lipids, unknown melanoidins, minerals, and alkaloids such as caffeine, trigonelline, and chlorogenic acid [1,2]. Non-volatile compounds degrade when they undergo thermal processing involving roasting. Chlorogenic acid and trigonelline are rapidly degraded during roasting, and phenolic compounds and pyridines/pyrroles are produced, respectively [3]. These newly formed compounds, along with other volatile compounds, influence the coffee quality or flavor.

Recently, considerable research has been conducted on coffee volatiles, especially the volatile compounds in hot coffee from an espresso machine [4] and coffee that undergoes different degrees of roasting at varying temperature and time [5], using headspace solid-phase microextraction (HS-SPME) coupled with gas chromatography—mass spectrometry (GC—MS). Other studies related to specific coffee beans have also been conducted. Cheong et al. [6] explored the volatile composition of Arabica coffee, and Agresti et al. [7] identified five volatile compounds in a defective Brazilian coffee beans, which could be utilized to discriminate between defective and non-defective Brazilian coffee beans. Among the diverse types of volatile compounds in coffee, volatile thiols were studied by Dulsat-Serra et al. [8]; they suggested that the amounts of volatile thiols in brewed coffee are affected by not only the innate amount in the coffee but also the extraction process. The variabilities in the volatile compound profiles of individual coffee beans were also analyzed [9].

Bhumiratana et al. [10] studied the aroma profiles of roasted, ground, and brewed coffee. They used three varieties of Arabica coffee—El Salvador Bourbon, Ethiopia Kebado, and Hawaii Kona—as samples and three roasting processes, namely light, medium, and dark. Their research showed that both origin and roasting condition of coffee yielded different coffee flavors, especially “burnt/acrid, pungent, ashy/sooty, and sour notes”. Cordoba et al. [11] also showed that the grinding and extraction time influenced the sensory perception of Colombia (Huila and Narino) coffee samples. Steen et al. [12] reported that the serving temperature, in addition to the grinding and brewing, was crucial to the quality of coffee. Six serving temperatures—31, 37, 44, 50, 56, and 62 °C—were used in this study. When compared with sensory data, 31-, 37-, and 44-°C coffee samples were related to the characteristics of sourness, tobacco, and sweetness, whereas higher serving temperatures—50, 56, and 62 °C—were related to the characteristics of bitterness, roasted, and overall intensity of coffee. They suggested that the serving temperature is related to certain volatile compounds that influence the flavor perception. According to Angeloni et al. [13], the extraction method (cold drip, cold brew (CB) coffee) and temperature also affected the caffeine and chlorogenic compounds. Although diverse factors, such as the origin, roasting condition, grinding degree, extraction time, serving temperature, and extraction methods of coffee, were considered in these studies, research on the effect of brewing temperature on sensory perception related to non-volatile and volatile compounds in CB coffee is limited. Considering that the flavor and aroma of coffee are affected by each step of the coffee-production process, CB coffee may show different compositions of non-volatile compounds and volatile profiles compared to hot extracted coffee. Thus, the compositions of volatile compounds of various CB coffee types need to be further explored. Hot extracted coffee and CB coffee are considered to have different patterns of volatile and non-volatile compounds; thus, they should be compared.

The objectives of this study are to (1) identify the volatile profiles of CB coffee, CB coffee from a coffee shop (CS), and ready-to-drink (RTD) CB coffee using HS-SPME—GC—MS; (2) quantify caffeine, chlorogenic acid, and trigonelline contents using high-performance liquid chromatography (HPLC); (3) determine the relationship between each compound and CB coffee samples based on semi-quantified volatile compounds; and (4) compare caffeine, trigonelline, chlorogenic acid, and volatile compound profiles of general hot extracted coffee with those of CB coffee.

## 2. Materials and Methods

### 2.1. Coffee Samples and Preparation

#### 2.1.1. Brewed Coffee

A total of 13 coffee samples were used. Blended, Kenya AA, and Colombia beans were used for brewed coffee (Table 1). All coffee beans were ground using a grinder (Delonghi KG79, Treviso, Italy) with a medium setting. Ground coffee, Folgers classic roast (Folgers, Orrville, OH, USA), was also included as a brewed coffee sample. The samples were brewed using methods including cold brewing and coffee maker (CM). These samples were obtained using 7 g of each ground coffee per 100 mL of water.

Ground coffee was first placed in a glass bottle (standard wide-mouth bottles with polyvinyl-lined caps, Fisher Scientific, Pittsburgh, PA, USA) for cold brewing, and bottled water (Samdasoo 2L, Kwang Dong Pharmaceutical. Co., Seoul, Korea) was poured over it. After 9 h of brewing at 4 °C, coffee was filtered through a stainless filter (stainless filter, 4 cups, Kinto, Hikone-shi, Shiga, Japan) and re-filtered through a paper filter (Mellitta paper filter 1 × 4, Mellitta, Minden, North Rhine-Westphalia, Germany) to remove the fine particles in it. The filtered brewed coffee was kept frozen until analysis.

A coffee maker (Phillips HD-7564, Amsterdam, The Netherlands) was used to make brewed coffee (CM); the coffee was brewed for 10 min, and a 1 × 4 paper filter was used for filtering (Mellitta paper filter, Mellitta, Minden, North Rhine-Westphalia, Germany). The CM brewed samples were cooled to room temperature (20 °C) and then kept frozen until the analysis.

#### 2.1.2. Ready-to-Drink (RTD) Coffee

RTD coffee samples—French cafe CB coffee (Namyang, Seoul, Korea) and Barista Rules CB coffee (Maeil, Seoul, Korea)—were purchased from a convenience store (GS retail, Busan, Korea). The CB coffee was delivered by Babinski coffee (Korea Yacult, Seoul, Korea). The samples were kept frozen until the instrumental analyses, and the sample information is provided in Table 1.

#### 2.1.3. Coffee from a Coffee Shop (CS)

CB coffee was purchased from nearby local Starbucks (Seattle, WA, USA) and Twosomeplace (CJ Foodville, Seoul, Korea) (Table 1). Both CB coffee samples were purchased without ice to prevent the dilution and kept frozen until the instrumental analyses.

### 2.2. Analysis of Caffeine, Chlorogenic Acid, and Trigonelline

#### HPLC

Caffeine, chlorogenic acid, and trigonelline were quantified in the CB samples. For clarifying, the coffee samples were treated with potassium ferrocyanidetrihydrate (Sigma-Aldrich Co., St. Louis, MO, USA), zinc acetate dehydrate (Sigma-Aldrich Co., St. Louis, MO, USA), and glacial acetic acid (Sigma-Aldrich Co., St. Louis, MO, USA) as Carrez reagents I and II. A total of 10 milliliters of coffee and 0.3 mL of Carrez reagents were mixed and centrifuged for 10 min at 45,000× *g* (CF15R, Hitachi Koki Co. Ltd., Tokyo, Japan). The supernatant was then filtered through a 0.45 μm membrane filter (Acrodisc Syringe Filter, Gelman Sciences, Ann Arbor, MI, USA). The column for caffeine analysis was a XBridge™ Shield RP18 (5 μm, 4.6 mm × 250 mm, Waters, Milford, MA, USA), mobile phase A was 10 mM citric acid, and mobile phase B was methanol (HPLC-grade, Merck, Damstadt, Frankfurter, Germany). The gradient mode was initially set at an A/B ratio of 85/15 from 0 to 10 min, which linearly increased to 60/40 at the 10 min mark and ended at the 30 min mark. Of each sample, 20 mL were injected, and the flow rate was adjusted to 1.0 mL/min. A UV detector (Shimadzu SPD-20A, Kyoto, Japan) was set at 276 nm. The concentration of caffeine was calculated using the peak area of a commercial standard (Sigma-Aldrich Co., St. Louis, MO, USA).

For chlorogenic-acid analysis, the coffee samples were preprocessed and treated in the same way as caffeine, but the detector was set at 325 nm. The concentration of chlorogenic acid was calculated using the peak area of a commercial standard (5-caffeoylquinic acid, Sigma-Aldrich Co., St. Louis, MO, USA).

Each 10 mL coffee sample was clarified with 0.3 mL of Carrezz reagents I and II and 0.6 mL of absolute methanol (HPLC-grade, Merck, Damstadt, Frankfurter, Germany) for trigonelline analysis. After the treatment, the same HPLC process for caffeine and chlorogenic-acid analyses was performed with the following changes. As a mobile phase, 0.5% methanol (HPLC-grade, Merck, Damstadt, Frankfurter, Germany) was used, and a reverse-phase column (XBridge™ Shield RP18; 5 μm, 4.6 mm × 150 mm, Waters, Milford, MA, USA) was also used. The flow rate was 0.8 mL/min and detected at 264 nm. The concentration of trigonelline was calculated using the peak area of a commercial standard (Sigma-Aldrich Co., St. Louis, MO, USA). Each non-volatile compound was analyzed for 5 replications.

### 2.3. Analysis of Volatile Compounds

#### 2.3.1. Headspace Solid-Phase Microextraction (HS-SPME)

In a 10 mL vial, 2 mL coffee samples were placed with a silicon/PTFE septum (18 mm (diameter) × 3.2 mm (thickness); Varian, Inc., Walnut Creek, CA, USA). Five microliters of 0.045 mg/mL 1,3-dichlorobezene (Sigma-Aldrich, St. Louis, MO, USA) solution (in methanol) was added to the sample as an internal standard for semi-quantification of the volatile compounds in the coffee samples. These coffee-sample vials were equilibrated for 10 min at 60 °C in an autosampler (Model GC Sampler 80, Agilent Technologies, Santa Clara, CA, USA) with agitation at 250 rpm. Next, a 50/30 µm 3-phase (DVB/CAR/PDMS) SPME fiber (Supelco, Bellefont, PA, USA) was used to collect the volatile compounds of the coffee samples at 60 °C for 45 min.

#### 2.3.2. Gas Chromatography—Mass Spectrometry (GC—MS)

After sampling, the fiber was inserted into the injection port of the GC—MS (Model 7890A, Agilent Technologies, Santa Clara, CA, USA) connected to a spectrophotometer detector (Model 5977A, Agilent Technologies, Santa Clara, CA, USA), and HP-5 ms column (30 m × 250 µm × 0.25 µm thickness, Agilent Technologies, Santa Clara, CA, USA) was used to separate the volatile compounds. A split-less mode was applied at 250 °C for 5 min. The initial temperature of the column was 40 °C for 10 min, increased by 8 °C/min to 180 °C and then by 10 °C/min to 280 °C, and it was maintained at 280 °C for 10 min. The volatile compounds in each sample were analyzed in triplicate. Each volatile compound was quantified as the mean concentration (ng/mL).

Most compounds were identified and compared using 2 different analytical methods: Kovats indices and mass spectra comparison using the National Institute of Standards and Technology (NIST) library of compounds (NIST/EPA/NIH mass spectral library, version 2.2, 2014). Semi-quantification of the volatile compound concentrations in each coffee sample was calculated and reported based on the internal standard concentration.

#### 2.3.3. Consumer Evaluation

Including 1 blind duplicate sample (BaristaRTD (A) and (B)), 14 samples were provided to the participants. CB and CM samples were extracted using 7 g of ground coffee with 100 mL of water, and 50 mL of each sample was provided in a plastic cup. Unsalted crackers and water were also provided for cleansing the palate. The evaluations were held over 2 sessions, and 120 consumers participated in the evaluations; the sensory liking and intensity of coffee samples were investigated. This consumer study was approved by the institutional review board of Pusan National University (IRB 2016_49_HR). Details of the consumer data analyses were available [14], and only the consumers’ acceptability data were used in this study to analyze their relationship with the constituents of coffee.

### 2.4. Statistical Analysis

Two-way analysis of variance (two-way ANOVA) was conducted to investigate the effects of the brewing methods, CB and CM, and coffee variety (brand), and their interaction, variety (brand) × brewing method. In addition, a one-way analysis of variance (one-way ANOVA) was conducted on RTD commercial coffee products, CS samples, and 8 samples of different brewing methods and coffee variety (brand). The significant difference (LSD) was also calculated post hoc. Pearson’s correlation analysis was conducted to explore the relationship between volatile compounds and the consumers’ perceived sensory intensity and overall liking. All these analyses were conducted using SAS 9.4 (SAS Institute Inc., Cary, NC, USA). To understand the relationship between coffee samples and volatile compounds, principal component analysis (PCA) was used. Internal preference mapping was also used to examine the relationship between coffee samples and components based on the consumers’ liking using XLSTAT 19 (Addinsoft, New York, NY, USA).

## 3. Results and Discussion

### 3.1. Caffeine, Chlorogenic Acid, and Trigonelline Compounds in CB Coffee Samples

Alkaloids, such as caffeine, chlorogenic acids, and trigonelline, are crucial to the coffee flavor [1]. Bitterness was one of the main sensory attributes influencing consumer acceptability [15]. Both caffeine and chlorogenic acid contributed to the bitterness, whereas the latter was also responsible for astringency [1,16].

*p*-values of caffeine, chlorogenic acid, and trigonelline contents in the CB and CM samples are listed in Table 2. The coffee variety (brand) and brewing method significantly affected these contents. The caffeine content was affected by both the coffee variety (brand) and brewing method, whereas the chlorogenic acid and trigonelline contents were significantly affected by the coffee variety.

The amounts of the three non-volatile compounds are shown in Table 3. Generally, the RTD samples had the lowest amounts of these compounds. The caffeine contents descended in the following order: Those of FolgersCM, StarbucksCS, FolgersCB, and TwosomeplaceCS. The contents of chlorogenic acid and trigonelline were the highest in the StarbucksCS and FolgersCM samples. The CB and CM samples, except the Folgers samples, generally showed moderate contents: 0.74–0.8 mg/mL (caffeine), 0.18–0.23 mg/mL (chlorogenic acid), and 0.19–0.27 mg/mL (trigonelline) (Table 3). Kim and Kim [17] studied the contents of caffeine, chlorogenic acid, and trigonelline using different extraction methods. They used Kenya AA coffee samples and steeped the coffee grounds for 9 h to obtain CB coffee. The contents of caffeine, chlorogenic acid, and trigonelline were 0.69 mg/mL, 0.26 mg/mL, and 0.41 mg/mL, respectively. Although they used 50 g of ground coffee from beans and 500 mL of water to obtain the coffee samples, the contents of non-volatile compounds (except trigonelline) were similar to those in this study. In addition, in Blending, Colombia, and Kenya coffee samples, BlendingCB and BlendingCM contained slightly lower amounts of chlorogenic acid. Fuller and Rao [18] analyzed the caffeine and 3-chlorogenic-acid (3-CGA) contents in coffee prepared from grounds with different degrees of roasting and granule sizes and reported that the roasting temperature could affect the 3-CGA content. However, there was no significant difference between cold and hot brewed coffee [18]. Given that the blending samples underwent a stronger roasting level (city roasting) than other samples (generally medium roasting), a higher roasting temperature might influence the degradation of chlorogenic acid, leading to a reduction in its amount in the final coffee samples.

The loss in caffeine content due to general roasting is very low unless the coffee bean undergoes extreme-dark roasting [3]. The caffeine contents of CM samples (Colombia, Folgers) were higher than those of the CB samples, showing that the caffeine contents in these coffee samples were influenced by the brewing method (CB and CM). Considering that the caffeine content was higher in the 22 °C coffee samples than in the 5 °C samples [13], irrespective of the extraction method (brewing and dripping), this difference in caffeine content between the CB and CM samples was considered to be due to the extraction temperature. Furthermore, grinding and brewing methods can influence the caffeine content [19]. Thus, the low caffeine content in RTD samples in this study may be explained by the different grinding levels and brewing times of different manufacturing companies.

Blended CB and CM samples underwent a higher intensity of roasting than that for the Colombia and Kenya samples, which may explain the lower chlorogenic acid contents in the CM samples. This result agrees with those of other studies (e.g., Farah et al. [20] and Kim and Park [3]). According to their studies, chlorogenic acid can be degraded (>90%) by thermal processing such as dark roasting, suggesting that the roasting time and temperature influence the chlorogenic-acid content in coffee products [21,22].

Trigonelline contributes to the overall aroma perception of brewed coffee [23]. Usually, it has a very weak bitter taste, but it degrades rapidly during the roasting process and produces impactful volatile compounds such as pyridines and pyrroles [3]. The lower chlorogenic-acid and trigonelline contents in RTD samples can possibly be ascribed to several thermal processes during manufacturing because both chlorogenic and trigonelline were vulnerable to thermal processes such as roasting. Degradation during processing could also be considered a reason for the reduced amount of chlorogenic acid in the RTD samples. Additionally, according to a study conducted by Andueze et al. [24], caffeine, chlorogenic acid, and trigonelline contents increased with a decrease in particle size of the espresso coffee samples. Furthermore, when ground coffee was exposed to heat or high temperature, some flavor loss occurred [25].

### 3.2. Volatile Compounds in CB Coffee Samples

A total of 36 volatile compounds, including phenolic compounds such as guaiacol, pyrazines, pyridines, and pyrroles, were identified (Table 4). The relative standard deviation (RSD, %) for each coffee sample and threshold of each volatile compound are listed in Table 5 and Table 6, respectively.

Pyridine (V1) is known to have bitter, burnt, roasted, and astringent characteristics [26,27], and it can give a sharp burnt taste at concentrations as low as those on the ppm scale [28]. In this aspect, only StarbucksCS (1229 ng/mL) and TwosomeplaceCS (1695 ng/mL) might have a burnt taste. Pyridine is produced by trigonelline degradation and Maillard reactions [29], especially in coffee that is subjected to high temperatures or strong roasting processes [5]. According to Bhumiratana et al. [10], the degree of roasting and preparation process contributed to aroma profiles rather than the coffee variety. Corresponding to this research, Blending CB and CM samples showed relatively higher contents of pyridine (694 and 643 ng/mL) than those in Colombia and Kenya samples of CB (521 and 508 ng/mL) and CM (268 and 309 ng/mL), respectively. As indicated by the sample sellers, the blending sample was roasted to the city level (about medium~dark roasting), and Colombia and Kenya beans were roasted to a medium level. Commercially ground coffee, Folgers, had a higher pyridine content, although it was indicated as a medium-roasted, and BaristaRTD had a higher pyridine content than those of other RTD samples. The CS samples had the highest amount of pyridine among all the evaluated coffee samples. In the case of 3-ethylpyridine (V6), which was correlated with a roasted, burnt aroma [27] and tobacco flavor [30], was found in TwosomeplaceCS and BaristaRTD at higher concentrations. N-acetyl-4-(H)-pyridine (V12) showed higher concentrations in CS and Folgers samples. The threshold of furfural (V3) was 3 ppm [31], which was significantly higher than the furfural contents of all the coffee samples in this study.

Main phenolic compounds such as guaiacol, 4-ethylguaiacol (V29), and 4-vinylguaiacol (V31) were also identified. The threshold of guaiacol and 4-vinylguaiacol (V31) was reported to be 3 ppb [26,31]. Guaiacol is related to smoke, sweet, and medicinal characteristics [32], and it evokes a burning sensation even if it exists at very low concentrations [28]. 4-ethylguaiacol (V29) and 4-vinylguaiacol (V31) are known to be associated with spice and clove flavors [32]. 4-ethylguaiacol (V29) is known to have a “spicy-medicinal” highly powerful odor [28]. Folgers samples showed higher contents of both 4-ethylguaiacol (V29) and 4-vinylguaiacol (V31), and the CS and Folgers samples had higher contents of guaiacol. Meanwhile, pyrazines are known to have roasted and earthy characteristics in ground coffee [1]. The contents of 2,6-dimethylpyrazine (V5) associated with nutty coffee odor [30], methylpyrazine (V2), 2-ethyl-3-methylpyrazine (V9), and 5H-5-methyl-6,7-dihydrocyclopenta[b]pyrazine (V25) were higher in the CS and Folgers samples. Folgers and TwosomeplaceCS samples had higher amounts of 2-ethyl-3,5-dimethylpyrazine (V18), which has roasted cocoa odor [25], 3-ethyl-2,5-dimethylepyrazine (V17), and 2,5-diethylpyrazine (V19) than those of the other samples.

Difurfuryl ether (V30) has a coffee-like and toasted odor [26], and BaristaRTD, CS, and Folgers samples had higher amounts of difurfuryl ether (V30) than the other samples. Folgers and CS samples contained higher amounts of 2-furanmethanol (V4) and 2-methyl-5-propionylfuran (V24). Phenol (V8), which is related to phenol materials [32] and smoky [26], burning taste, coal tar odor [25], also showed higher amounts in CS and Folgers samples.

Pyrroles are usually related to nutty, hay-like, and herb aroma. First, 1-Furfuryl pyrrole (V26), which has hay-like, mushroom, and green characteristics [26], was higher in FolgersCM. In the case of 2-acetylepyrrole (V14), CS and Folgers samples showed higher contents. With respect to tetradecanoic acid (V36), which was reported to be associated with faint odor [30], only ColombiaCB and KenyaCB contained about 1 ng/mL. The concentration of 3,5-octadien-2-one (V15) was higher in ColombiaCB and KenyaCB than in other samples.

Maltol (V23) is associated with caramel-like odor and chocolate flavor [30]. When in solution, it exhibits fruity characteristics, especially when the concentration is below 20 ppm [28]. StarbucksCS had a higher amount of maltol, followed by TwosomeplaceCS, ColombiaCM, and KenyaCM showed lower contents than those of ColombiaCB and KenyaCB. These volatile compound patterns suggested that the Colombia and Kenya samples were more influenced by the extraction method than the origin of coffee. Fisk et al. [33] compared the contents of aroma compounds in brewed coffee. Among the key aroma compounds that they reported, guaiacol (V20) and maltol (V23) were identified in our coffee samples. In their study, guaiacol (V20) and maltol (V23) contents detected by LLE—GC—MS were higher in the Kenya brewed coffee sample than in the Colombia brewed coffee sample. Similarly, Kenya samples had higher amounts of guaiacol (V20) and maltol (V23) than those in Colombia samples, regardless of the extraction method (CB and CM) in this study. This result might be due to the difference in coffee bean components that affect volatile compounds rather than the difference in extraction methods.

Nonanal (V22) is related to citrus, fat, and green attributes [32]. KenyaCB (2 ng/mL), ColombiaCB and CM (0 and 3 ng/mL, respectively), FrenchCafeRTD (3 ng/mL), and BabinskiRTD (2 ng/mL) are more likely to have a refreshing, citrusy, and waxy taste because n-nonanal showed these characteristics when it was below a concentration of 5 ppm [28]. Nonanoic acid (V28) is also related to green, fatty, and bitterness, and this compound was found in higher concentrations in FolgersCM and StarbucksCS. Decanoic acid (V32), which had rancid fatty notes [28] were present in slightly higher amounts in FolgerCM and StarbucksCS samples. Although there were no peppermint-like attributes, ColombiaCB and FolgersCB showed slightly higher amounts of methyl salicylate (V27), which is associated with peppermint characteristics [32].

The results of volatile compounds showed that CS and Folgers samples had higher amounts of volatiles than those in RTD samples. Specifically, RTD samples, except BaristaRTD, generally had lower contents of most volatile compounds, which corresponded with non-volatile compounds, showing the lowest contents in RTD samples. Kumazawa and Masuda [34] suggested that heat treatment for sterilization could affect the sensory aspect of canned coffee. The results of sensory evaluation showed significant differences between heated coffee and non-heated coffee samples. Furthermore, after heat treatment, 3-methylbutanoic acid, and another “unknown” compound related to sour odor were identified by gas chromatography-olfactometry (GC-O). Although coffee volatiles that were affected by heat processing might vary according to coffee variety or specific brewing preparation, some volatile compounds of RTD could change. Amanpour and Selli [35] compared the concentrations of volatile compounds in Turkish coffee and French press coffee. The results showed that volatile profiles of both coffee samples were similar, but there were still a few differences in the volatile compounds and concentrations. This research suggests that the brewing method could influence volatile profiles. In a study of volatile compounds in brewed green teas [36], the authors mentioned that there was a possibility of temperature influencing the threshold of volatile compounds.

**Table 4:** Concentration of volatile compounds of coffee samples (ng/mL) ^1^.

RT ^2^	No.	Compounds	Base Peak (*m/z* ± 0.5 amu)	KI (Lit) ^3^	KI (exp)	BlendingCB	ColombiaCB	Kenya CB	Folgers CB	BlendingCM	ColombiaCM	Kenya CM	Folgers CM	Barista RTD	BabinskiRTD	French cafeRTD	Starbucks CS	TwosomeplaceCS
4.105	V1	Pyridine	79	769 [37]	787	694 ± 50.6	521 ± 62.5	508 ± 61.3	870 ± 110	643 ± 241	268 ± 20.5	309 ± 32.4	815 ± 76.2	972 ± 76.1	515 ± 42.2	772 ± 53.2	1229 ± 73.9	1695 ± 185
7.015	V2	Methylpyrazine	94	826 [38]	817	62 ± 6.54	85 ± 15.9	72 ± 14.4	149 ± 17.9	62 ± 24.6	42 ± 6.40	41 ± 6.82	156 ± 14.6	45 ± 2.91	69 ± 5.26	43 ± 2.85	116 ± 6.33	98 ± 11.5
7.500	V3	Furfural	96	830 [39]	826	120 ± 8.50	354 ± 65.3	309 ± 41.0	105 ± 11.0	116 ± 45.0	156 ± 34.6	169 ± 37.6	113 ± 9.11	32 ± 1.90	74 ± 4.91	44 ± 3.42	94 ± 6.30	50 ± 5.27
9.126	V4	2-Furanmethanol	98	853 [40]	853	82 ± 8.06	101 ± 21.7	96 ± 18.2	127 ± 17.9	86 ± 35.1	44 ± 7.88	53 ± 10.4	136 ± 15.5	74 ± 3.92	71 ± 6.79	60 ± 3.71	191 ± 11.8	137 ± 18.8
12.39	V5	2,6-Dimethylpyrazine	108	910 [41]	913	125 ± 15.1	164 ± 30.3	137 ± 24.5	229 ± 27.1	120 ± 47.3	85 ± 11.9	75 ± 10.9	235 ± 18.9	81 ± 4.62	104 ± 6.58	84 ± 5.36	187 ± 11.7	173 ± 19.5
14.34	V6	3-Ethylpyridine	92	959 [42]	963	23 ± 4.51	9 ± 1.65	12 ± 1.76	45 ± 6.67	17 ± 6.86	3 ± 0.32	5 ± 0.69	37 ± 3.12	76 ± 9.90	11 ± 1.11	52 ± 4.22	61 ± 6.51	111 ± 12.9
14.57	V7	5-Methyl-2-furaldehyde	110	966 [43]	969	183 ± 17.1	323 ± 55.6	317 ± 43.2	166 ± 17.8	183 ± 72.4	145 ± 36.1	178 ± 37.9	178 ± 14.8	52 ± 4.38	99 ± 7.02	76 ± 4.70	157 ± 8.72	103 ± 11.0
15.32	V8	Phenol	94	989 [44]	988	9 ± 1.07	8 ± 1.57	10 ± 1.29	24 ± 3.15	10 ± 3.71	4 ± 0.96	6 ± 1.35	29 ± 2.39	19 ± 1.52	7 ± 0.57	15 ± 1.22	27 ± 1.65	39 ± 4.41
15.71	V9	2-Ethyl-3-methylpyrazine	121	999 [38]	998	61 ± 10.4	66 ± 11.8	57 ± 10.7	108 ± 12.3	51 ± 20.7	25 ± 4.23	25 ± 3.8	105 ± 7.39	38 ± 2.41	33 ± 2.14	44 ± 2.00	69 ± 4.68	87 ± 9.09
16.07	V10	Pyrrole-2-carboxaldehyde	95	1008 [45]	1010	39 ± 5.58	47 ± 10.0	53 ± 8.95	42 ± 6.27	41 ± 15.0	18 ± 4.88	27 ± 5.62	46 ± 4.03	19 ± 7.60	17 ± 1.69	28 ± 2.11	49 ± 2.43	53 ± 5.05
16.24	V11	1-Methyl-1H-pyrrole-2-acetonitrile	120	1118 [46]	1017	6 ± 0.90	8 ± 1.61	7 ± 1.11	9 ± 0.92	5 ± 1.91	3 ± 0.56	3 ± 0.49	9 ± 0.57	3 ± 0.21	3 ± 0.18	3 ± 0.20	6 ± 0.45	6 ± 0.64
16.38	V12	N-acetyl-4(H)-pyridine	80	1038 [47]	1022	5 ± 0.58	5 ± 0.79	4 ± 0.62	10 ± 1.42	5 ± 1.98	2 ± 0.47	3 ± 0.50	11 ± 1.09	5 ± 0.30	4 ± 0.09	2 ± 0.12	10 ± 0.50	8 ± 0.92
17.36	V13	3-Methylphenol	108	1065 [48]	1059	3 ± 0.32	2 ± 0.34	3 ± 0.25	4 ± 0.37	4 ± 1.56	1 ± 0.27	2 ± 0.59	5 ± 0.42	4 ± 0.36	8 ± 0.83	4 ± 0.35	5 ± 0.44	7 ± 0.64
17.49	V14	2-Acetylpyrrole	94	1060 [49]	1063	28 ± 4.77	27 ± 6.39	29 ± 5.47	44 ± 7.72	31 ± 11.9	12 ± 3.73	15 ± 3.84	50 ± 4.64	32 ± 2.75	16 ± 1.96	24 ± 2.15	53 ± 2.64	67 ± 9.84
17.55	V15	3,5-Octadien-2-one	95	1081 [50]	1066	7 ± 0.69	19 ± 4.00	17 ± 2.80	6 ± 0.82	8 ± 3.11	8 ± 2.09	9 ± 1.95	8 ± 0.74	2 ± 1.60	4 ± 0.48	3 ± 0.14	8 ± 0.59	4 ± 0.69
17.82	V16	2-Acetyl-1-methylpyrrole	108	1096 [51]	1076	19 ± 2.42	15 ± 2.29	16 ± 2.36	24 ± 2.54	18 ± 7.89	6 ± 1.36	8 ± 2.00	27 ± 2.20	20 ± 1.83	1 ± 0.11	20 ± 1.31	28 ± 2.08	39 ± 3.52
17.95	V17	3-Ethyl-2,5-dimethylpyrazine	135	1078 [38]	1081	84 ± 15.1	88 ± 16.7	65 ± 12.4	154 ± 16.3	63 ± 24.8	29 ± 4.48	25 ± 3.10	130 ± 8.25	44 ± 3.96	30 ± 2.40	58 ± 3.77	68 ± 4.77	112 ± 12.1
18.09	V18	2-Ethyl-3,5-dimethylpyrazine	135	1085 [52]	1086	12 ± 1.82	12 ± 2.57	10 ± 1.77	23 ± 2.44	10 ± 3.86	4 ± 0.59	4 ± 0.41	20 ± 1.00	8 ± 0.40	5 ± 0.57	9 ± 0.57	12 ± 0.85	18 ± 2.37
18.14	V19	2,5-Diethylpyrazine	121	1085 [53]	1088	14 ± 3.18	14 ± 2.91	12 ± 2.26	29 ± 3.16	11 ± 4.62	5 ± 0.90	5 ± 0.83	26 ± 2.20	9 ± 0.84	6 ± 0.48	10 ± 0.76	14 ± 1.51	21 ± 2.04
18.18	V20	Guaiacol	109	1090 [43]	1089	15 ± 1.80	14 ± 2.18	16 ± 2.01	46 ± 5.32	15 ± 5.84	6 ± 1.89	8 ± 1.85	54 ± 4.33	26 ± 2.54	10 ± 1.04	27 ± 2.05	41 ± 2.32	57 ± 5.21
18.24	V21	3-Ethyl-2-hydroxy-2-cyclopenten-1-one	126	1091 [54]	1092	5 ± 0.49	7 ± 1.10	7 ± 1.08	6 ± 0.68	5 ± 2.03	3 ± 0.66	4 ± 1.09	7 ± 0.56	3 ± 0.38	3 ± 0.25	4 ± 0.23	9 ± 0.67	7 ± 0.49
18.55	V22	Nonanal	57	1102 [55]	1104	8 ± 1.87	0 ± 0.00	2 ± 0.16	8 ± 2.67	9 ± 2.32	3 ± 0.74	7 ± 1.65	7 ± 5.48	7 ± 1.85	2 ± 0.48	3 ± 1.17	7 ± 1.84	7 ± 3.79
18.71	V23	Maltol	126	1139 [47]	1111	20 ± 1.87	22 ± 5.54	29 ± 7.30	20 ± 5.94	25 ± 7.53	9 ± 5.16	11 ± 3.47	21 ± 3.62	21 ± 1.12	13 ± 3.43	24 ± 4.67	65 ± 5.31	34 ± 10.0
19.14	V24	2-Methyl-5-propionylfuran	109	1151 [56]	1131	12 ± 1.69	12 ± 2.07	14 ± 2.08	13 ± 1.26	11 ± 4.54	4 ± 1.31	7 ± 1.76	13 ± 1.30	11 ± 1.31	10 ± 0.82	12 ± 0.73	18 ± 2.22	24 ± 2.60
19.36	V25	5H-5-methyl-6,7-dihydrocyclopenta[b]pyrazine	119	1147 [57]	1141	6 ± 1.12	7 ± 1.74	6 ± 1.12	15 ± 1.95	6 ± 2.32	3 ± 0.78	3 ± 0.54	16 ± 0.97	6 ± 0.52	3 ± 0.39	5 ± 0.36	9 ± 0.70	13 ± 1.62
20.26	V26	1-Furfurylpyrrole	81	1189 [47]	1181	11 ± 1.34	9 ± 0.61	8 ± 2.35	17 ± 1.82	18 ± 6.65	3 ± 0.93	5 ± 1.28	27 ± 3.45	15 ± 3.39	1 ± 0.12	2 ± 0.19	8 ± 0.35	8 ± 1.82
20.51	V27	Methyl salicylate	120	1197 [58]	1195	230 ± 50.2	315 ± 72.5	252 ± 32.6	313 ± 30.4	228 ± 89.0	248 ± 32.3	255 ± 50.8	252 ± 61.4	266 ± 39.6	228 ± 36.5	219 ± 49.5	299 ± 20.7	275 ± 76.5
21.86	V28	Nonanoic acid	60	1270 [59]	1265	47 ± 15.4	4 ± 1.24	10 ± 5.90	41 ± 16.8	45 ± 35.4	4 ± 2.43	28 ± 8.93	82 ± 66.2	57 ± 72.2	5 ± 1.08	3 ± 2.27	83 ± 48.5	29 ± 12.9
22.12	V29	4-Ethylguaiacol	137	1280 [60]	1279	31 ± 5.44	25 ± 4.22	23 ± 5.03	173 ± 22.9	36 ± 14.9	11 ± 3.01	13 ± 2.28	207 ± 13.9	79 ± 10.9	13 ± 2.28	38 ± 4.09	72 ± 8.42	89 ± 10.3
22.53	V30	Difurfuryl ether	81	1305 [47]	1300	31 ± 3.26	18 ± 1.61	27 ± 6.46	50 ± 6.41	40 ± 17.4	7 ± 3.43	14 ± 6.03	60 ± 3.75	61 ± 11.6	6 ± 1.26	28 ± 4.48	54 ± 3.83	66 ± 9.24
22.75	V31	4-Vinylguaiacol	135	1315 [43]	1313	10 ± 1.37	30 ± 4.89	14 ± 3.72	35 ± 6.72	20 ± 8.11	22 ± 5.60	14 ± 3.53	76 ± 9.10	3 ± 0.60	1 ± 0.26	2 ± 0.28	5 ± 0.22	3 ± 0.92
23.56	V32	Decanoic acid	60	1362 [61]	1360	2 ± 0.19	0 ± 0.00	3 ± 1.79	3 ± 1.37	5 ± 4.23	1 ± 0.26	3 ± 1.34	6 ± 3.67	2 ± 2.02	1 ± 0.23	1 ± 0.37	6 ± 1.09	2 ± 0.73
23.64	V33	3,4-Dimethoxystyrene	164	1365 [61]	1365	6 ± 0.85	6 ± 0.97	10 ± 3.09	6 ± 0.92	9 ± 4.27	3 ± 1.30	7 ± 2.28	7 ± 0.44	4 ± 0.54	1 ± 0.33	3 ± 0.47	8 ± 0.76	5 ± 0.59
25.96	V34	2,4-Di-tert-butylphenol	191	1513 [62]	1507	88 ± 31.8	58 ± 5.07	81 ± 42.2	113 ± 20.9	79 ± 33.0	83 ± 8.84	89 ± 17.2	86 ± 27.4	90 ± 12.7	64 ± 1.49	92 ± 22.1	101 ± 23.0	119 ± 49.1
26.65	V35	Dodecanoic acid	73	1566 [63]	1553	2 ± 0.71	4 ± 0.43	4 ± 0.38	3 ± 1.06	3 ± 0.66	3 ± 0.98	5 ± 2.48	4 ± 0.58	2 ± 0.25	3 ± 1.27	3 ± 0.52	6 ± 2.68	3 ± 1.63
29.37	V36	Tetradecanoic acid	73	1756 [64]	1753	0 ± 0.00	1 ± 0.16	1 ± 0.11	0 ± 0.00	0 ± 0.00	0 ± 0.05	0 ± 0.00	0 ± 0.00	0 ± 0.00	0 ± 0.21	0 ± 0.05	0 ± 0.00	0 ± 0.00

^1^ Values stands for relative abundances expressed as ng/mL over the IS abundance. ^2^ RT: Retention time. ^3^ Each reference for KI was included.

**Table 5:** The relative standard deviation as a percent for the IS (1,3-Dichlorobenzene) peak abundance (average of three repetitions) for each coffee sample.

Sample	Relative Standard Deviation (%)
BlendingCB	5.7
ColombiaCB	7.6
KenyaCB	11.4
FolgersCB	14.0
BlendingCM	4.6
ColombiaCM	9.1
KenyaCM	8.2
FolgersCM	7.1
BaristaRTD	14.3
BabinskiRTD	7.5
FrenchCafeRTD	9.6
StarbucksCS	6.1
TwosomeplaceCS	10.3

**Table 6:** Sensory descriptors and odor threshold value of volatile compounds of coffee samples.

No	Volatile Compounds	Descriptors	Threshold (ng/mL)
V1	Pyridine	Sour, Fishy, Burnt [65], Smoky [35]	2000 [66]
V2	Methylpyrazine	-	-
V3	Furfural	Sweet, Wood, Almond [65]	3 [31]
V4	2-Furanmethanol	Sweet, Caramel, Coffee [65]	-
V5	2,6-Dimethylpyrazine	Cocoa, Roasted, Nutty [65]	1.72 [67]
V6	3-Ethylpyridine	Tobacco, Lethery [65]	-
V7	5-Methyl-2-furaldehyde	Caramel, Coffee [65]	-
V8	Phenol	Phenolic [65]	2400 [66]
V9	2-Ethyl-3-methylpyrazine	Nutty, Peanut, Musty, Earthy [65]	0.035 [67]
V10	Pyrrole-2-carboxaldehyde	Musty, Coffee [65]	-
V11	1-methyl-1H-pyrrole-2-acetonitrile	-	-
V12	N-acetyl-4(H)-pyridine	-	-
V13	3-Methylphenol	Phenolic [65]	31–800 [68]
V14	2-Acetylpyrrole	Musty, Nutty [65]	-
V15	3,5-Octadien-2-one	Fruity, Fatty [65]	-
V16	2-Acetyl-1-methylpyrrole	Earthy [65]	-
V17	3-Ethyl-2,5-dimethylpyrazine	Nutty [65]	1 [66]
V18	2-Ethyl-3,5-dimethylpyrazine	Almond, Burn, Coffee, Nutty [65]	0.04 [69]
V19	2,5-Diethylpyrazine	Sweet [67]	0.0017 [67]
V20	Guaiacol	Phenolic [65,70], Smoky [65], Burnt [35]	0.003 [26]
V21	3-Ethyl-2-hydroxy-2-cyclopenten-1-one	Caramel, Sweet [65]	-
V22	Nonanal	Waxy, Rose, Fresh [65]	0.001 [31]
V23	Maltol	Caramel, Sweet [65]	20 [26], 5800 [66]
V24	2-Methyl-5-propionylfuran	-	-
V25	5H-5-methyl-6,7-dihydrocyclopenta[b]pyrazine	Earthy, Rosted [65]	-
V26	1-Furfurylpyrrole	Vegetable, Green, Waxy [65]	-
V27	Methyl salicylate	Wintergreen, Mint [65]	0.04 [31]
V28	Nonanoic acid	Waxy, Cheese [65]	-
V29	4-Ethylguaiacol	Spicy [65,70], Sweet [35]	35 [71]
V30	Difurfuryl ether	Coffee, Earthy, Mushroom [65]	-
V31	4-Vinylguaiacol	Woody, Dry, Roasted, Clove-like [35]	80 [71]
V32	Decanoic acid	Rancid, Sour, Fatty [65]	-
V33	3,4-Dimethoxystyrene	Green, Floral [65]	-
V34	2,4-Di-tert-butylphenol	-	-
V35	Dodecanoic acid	Fatty [65]	-
V36	Tetradecanoic acid	Waxy, Fatty [65]	-

### 3.3. Consumers’ Perceptions of Coffee Samples

*p*-Values for the CB and CM samples are listed in Table 7. Table 7 and results of mean intensity of consumers’ overall liking, bitterness intensity, and coffeeID intensity (Table 8) showed that the variety × brewing method effect and variety (brand) effect were significant in all categories (overall liking, bitterness intensity, and coffeeID intensity). FolgersCB was higher than FolgersCM in overall liking. Coffee variety (brand) significantly affected coffeeID intensity more than the brewing method.

### 3.4. Relationship between Coffee Acceptability and Volatile Compounds

**Figure 1:**
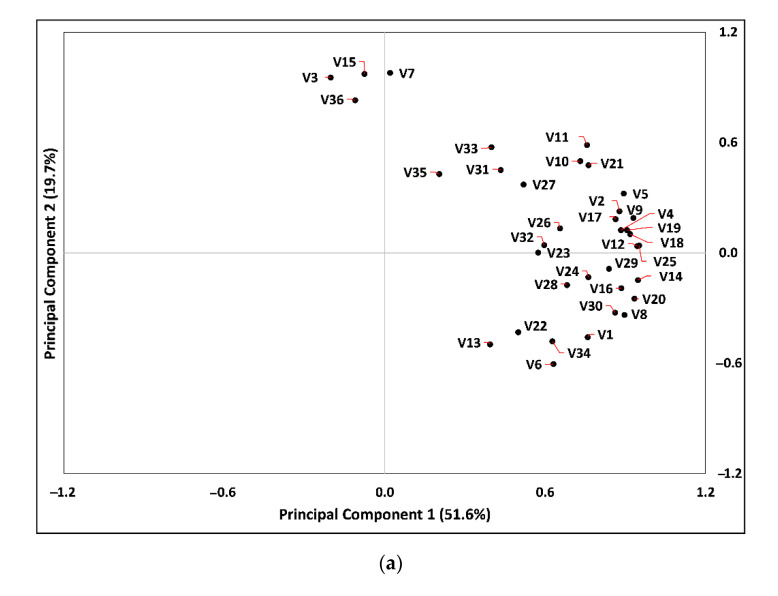

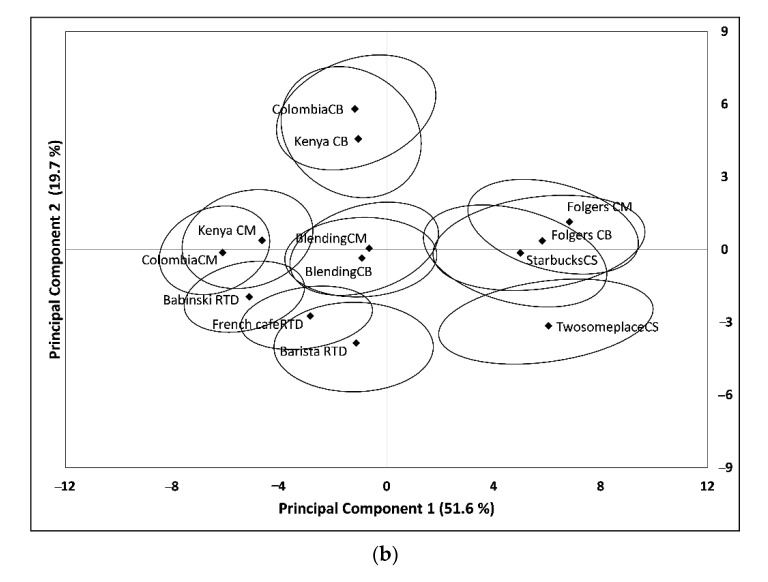
(**a**) Principal component analysis (PCA) of coffee samples and volatile compounds and (**b**) confidence ellipses map of the samples. V1–V36 stands for each volatile compound numbering in Table 4.

Volatile compounds in the coffee samples were investigated by PCA (Figure 1a,b). StarbucksCS, TwosomeplaceCS, and Folgers samples were positioned in quadrants 1 or 4, and most volatile compounds were also placed close to CS and Folgers samples. The CB and CM samples were positioned close together by extraction method rather than coffee variety. ColombiaCB and KenyaCB were related to 3,5-octadien-2-one (V15), 5-methyl-2-furaldehyde (V7), furfural (V3), and tetradecanoic acid (V36). ColombiaCM and KenyaCM were not related to any particular volatile compounds. This suggests that different extractions could influence volatile compound profiles markedly. However, blended coffees, BlendingCB and CM, were placed close to each other, showing that they were not different regardless of the extraction method. RTD samples were arranged in quadrant 3, and only BaristaRTD samples were slightly related to 3-methylphenol (V13) and nonanal (V22). The overall result of the confidence ellipse explained that sample discrimination by the brewing method was in the Colombia and Kenya samples. Furthermore, CS samples and Folgers samples were clearly discriminated between Colombia, Kenya, and RTD samples by volatile compounds.

### 3.5. Relationship between Sensory Data, Volatiles, and Caffeine, Chlorogenic Acid, and Trigonelline Compounds in Coffee

#### 3.5.1. Relationship between Sensory Data and Volatile Compounds

Overall, liking was negatively correlated with furfural (V3, *r* = −0.64), 5-methyl-2-furaldehyde (V7, *r* = −0.67), 4-vinylguaiacol (V31, *r* = −0.60), and only 3-methylphenol (V13, *r* = 0.62) was positively correlated with overall liking ratings as a result of correlation analysis (Table 9). This result seemed to be due to the absence of strong flavors induced by volatile compounds, affecting the overall liking of consumers. Bitterness intensity was correlated with many volatile compounds such as pyrazines (methylpyrazine, V2, *r* = 0.81; 2,6-dimethylpyrazine, V5, *r* = 0.80; 2-ethyl-3-methylpyrazine, V9, *r* = 0.68; 3-ethyl-2,5-dimethylpyrazine, V17, *r* = 0.66; 2-ethyl-3,5-dimethylpyrazine, V18, *r* = 0.69; 2,5-diethylpyrazine, V19, *r* = 0.72; and 5H-5-methyl-6,7-dihydrocyclopenta[b]pyrazine, V25, *r* = 0.79), pyrroles (1-methyl-1H-pyrrole-2-acetonitrile, V11, *r* = 0.63; 2-acetylpyrrole, V14, *r* = 0.72; and 2-acetyl-1-methylpyrrole, V16, *r* = 0.63), and guaiacols (guaiacol, V20, *r* = 0.74; 4-ethylguaiacol, V29, *r* = 0.74; and 4-vinylguaiacol, V31, *r* = 0.62), known to have roasted [1] nutty [26], smoke, and medicinal characteristics [32], respectively. Phenols (phenol, V8) and 2,4-di-tert-butylphenol (V34), which had burning and coal tar characteristics [32], were related to coffee ID intensity. Pyridine (V1, *r* = 0.63) and pyrroles (2-acetylpyrrol, V14, *r* = 0.71; 2-acetyl-1-methylpyrrole, V16, *r* = 0.68) were positively correlated with coffee ID intensity. The strong perception of coffee flavor might be influenced by volatile compounds, which have strong characteristics.

#### 3.5.2. Internal Preference Mapping

**Figure 2:**
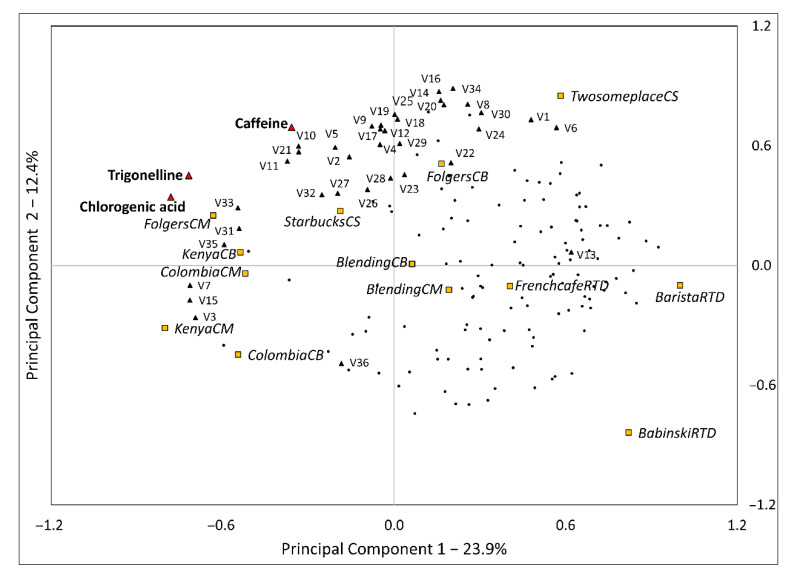
Internal preference map using consumers’ liking, coffee volatile and caffeine, chlorogenic acid, trigonelline, and coffee samples. V1–V36 stands for each volatile compound numbering in Table 4.

Figure 2 is an internal preference map based on consumers’ preference patterns, and it explained 36.3% of the variability for the first two principal components. This low variability seemed to be due to various factors. Most consumers liked RTD samples and were located in quadrants 1 and 4, whereas most volatile compounds were distributed mainly over quadrants 1 and 2. 3-methylphenol (V13) was positively related to consumers’ liking as an exception. Colombia and Kenya samples were placed in quadrants 2 and 3, the opposite side of consumers’ preference. Pyrazines were located on positive side of PC2 and two CS samples and FolgersCB were separated from others. From the PCA, pyridines isolated TwosomeplaceCS from all coffee samples. Aldehydes were grouped on PCA. As explained in 3.4. (Figure 1), Blending samples were displayed around the center of axis and CS samples, and Folgers samples were related to volatile compounds. This tendency explains that generally, lower concentrations of volatile compounds might positively affect consumers’ liking.

Coffee quality and its volatile compounds can be greatly influenced by coffee species, growing altitude, presence or absence of defects, postharvest processing such as washing and drying, roasting, and even storage conditions [72]. Moreover, synergistic effects among volatile compounds should also be considered when exploring volatile compound profiles. For example, maltol can be enhanced by vanillin and heliotropine [28]. Meanwhile, it appeared that the synergistic effect of volatile compounds existed according to the study of Ito and Kubota [73]. 4-Hexanolide was added to jasmine tea samples, and even though it was under the threshold level, it had an impact on sweet and astringent odor perception in the descriptive analysis. Thus, further studies could be conducted linking volatile compounds and their synergistic impact on consumers or a descriptive panel’s perception of coffee characteristics.

## 4. Conclusions

This study revealed the caffeine, chlorogenic acid, trigonelline, and volatile compound profiles of CB coffee. Typically, the amounts of both non-volatile and volatile compounds in CS and Folgers samples were higher than those in the RTD and CB samples. A comparison between CB and CM samples indicated that some volatiles were influenced by the brewing method. A comparison between the CB and CM samples did not reveal any consistent patterns of caffeine, chlorogenic acid, or trigonelline compounds. However, the caffeine content was higher in the CM samples than in the CB samples owing to the different brewing methods and extraction temperatures. The results of the correlation analysis suggested that particular pyrazines, pyrroles, and guaiacols were related to the bitterness intensity, and phenols were correlated with the coffeeID intensity. Overall, the RTD samples showed little correlation with volatile compounds and caffeine, chlorogenic acid, and trigonelline contents, whereas the CS and some CM samples (Colombia and Folgers) showed correlations with the volatile compounds and caffeine, chlorogenic acid, and trigonelline contents. Further studies on more RTD and CS samples should be conducted to gain a comprehensive understanding of CB coffee.

## Figures and Tables

**Table 1:** Coffee-sample information.

Coffee Samples	Variety	Degree of Roasting	Company or Purchasing Place
*Brewed coffee (CB, CM)*			
Blending	Brazil, Colombia, Ethiopia aricha, Guatemala	City	Coffee loves him (Busan, Korea)
Colombia Nariono Pasto Excelso		Medium	Koozeberry (Busan, Korea)
Folgers classic roast		Medium	Lotte Mart (Seoul, Korea)
Kenya AA Kagumoini		Medium	Coffee loves him (Busan, Korea)
*Ready-to-drink coffee*			
Barista rules cold brew	El Salvador SHG	Medium	Maeil (Seoul, Korea)
Cold brew by Babinski	Brazil, Colombia, Ethiopia	Between medium and high	Korea Yacult (Seoul, Korea)
French café cold brew	Ethiopia (Yergacheffe, Sidamo blend)	Unknown	Namyang (Seoul, Korea)
*Coffee from a coffee shop*			
Starbucks	Latin America and Africa blend	Medium	Starbucks (Seattle, WA, USA)
Twosomeplace	Unknown	Unknown	Twosomeplace (Seoul, Korea)

**Table 2:** Effects of coffee variety (brand) and brewing method on the *p*-values of caffeine, chlorogenic acid, and trigonelline contents in cold brew (CB) and coffee maker (CM) samples.

	*p*-Value		
	Variety (Brand) ^1^	Brewing Method ^2^	Variety × Brewing Method
Caffeine	<0.0001	0.0011	0.0008
Chlorogenic acid	<0.0001	0.4740	0.0070
Trigonelline	<0.0001	0.4970	0.0094

^1^ Varieties (Brands): Blending, Colombia, Folgers, and Kenya. ^2^ Brewing methods: CB and CM.

**Table 3:** Mean amount of caffeine, chlorogenic acid, and trigonelline of CB coffee samples (mg/mL) ^1,2,3^.

Sample Name	Caffeine	Chlorogenic Acid	Trigonelline
BlendingCB	0.80 ± 0.04 ^d^	0.21 ± 0.03 ^c,d,e^	0.25 ± 0.03 ^d^
ColombiaCB	0.63 ± 0.03 ^f^	0.21 ± 0.01 ^d,e^	0.25 ± 0.03 ^d^
FolgersCB	0.95 ± 0.04 ^b^	0.25 ± 0.02 ^a,b^	0.29 ± 0.02 ^b,c^
KenyaCB	0.75 ± 0.02 ^d,e^	0.23 ± 0.01 ^a,b,c^	0.28 ± 0.02 ^b,c,d^
BlendingCM	0.77 ± 0.07 ^d,e^	0.18 ± 0.02 ^f,g^	0.19 ± 0.03 ^e^
ColombiaCM	0.74 ± 0.04 ^e^	0.23 ± 0.01 ^b,c,d^	0.27 ± 0.03 ^c,d^
FolgersCM	1.05 ± 0.04 ^a^	0.25 ± 0.02 ^a^	0.31 ± 0.04 ^a,b^
KenyaCM	0.76 ± 0.03 ^d,e^	0.23 ± 0.01 ^b,c,d^	0.27 ± 0.03 ^c,d^
BabinskiRTD	0.63 ± 0.06 ^f^	0.16 ± 0.02 ^g,h^	0.18 ± 0.03 ^e^
BaristaRTD	0.59 ± 0.04 ^f^	0.10 ± 0.01 ^i^	0.11 ± 0.02 ^f^
FrenchCafeRTD	0.61 ± 0.03 ^f^	0.13 ± 0.01 ^h^	0.14 ± 0.02 ^f^
StarbucksCS	0.97 ± 0.04 ^b^	0.25 ± 0.03 ^a^	0.33 ± 0.01 ^a^
TwosomeplaceCS	0.87 ± 0.03 ^c^	0.19 ± 0.04 ^e,f^	0.26 ± 0.05 ^c,d^
*p*-value	<0.0001	<0.0001	<0.0001
LSD	0.05	0.03	0.04

^1^ CB stands for cold brew; CM stands for coffee maker brewed (hot); RTD stands for ready-to-drink; CS stands for coffee shop samples. ^2^ Mean ± SD. ^3^ Same characters in the same column mean no significant difference by ANOVA at 95% confidence level.

**Table 7:** *p*-Values of coffee variety (brand) and brewing method effects for consumers’ overall liking, bitterness intensity, and coffeeID intensity of CB and CM samples.

	*p*-Value		
	Variety (Brand) ^1^	Brewing Method ^2^	Variety × Brewing Method
Overall Liking	<0.0001	0.0149	0.0071
Bitterness Intensity	<0.0001	<0.0001	0.0202
CoffeeID Intensity	0.0041	0.1240	0.0073

^1^ Variety (Brand) stands for Blending, Colombia, Folgers, and Kenya. ^2^ Brewing methods stands for CB, CM.

**Table 8:** Mean intensity of consumers’ overall liking, bitter intensity, and coffeeID intensity of coffee samples.

Sample Name ^1^	Overall Liking ^2^	Bitterness Intensity ^2^	CoffeeID Intensity ^2^
BlendingCB	4.29 ± 1.97 ^b,c,d^	4.89 ± 1.75 ^d^	5.63 ± 1.53 ^b,c,d^
BlendingCM	4.56 ± 2.00 ^b,c,d^	4.89 ± 1.78 ^d^	5.21 ± 1.37 ^e,f^
ColombiaCB	3.64 ± 1.83 ^e,f,g^	4.60 ± 1.94 ^d,e^	5.20 ± 1.74 ^e,f^
ColombiaCM	3.50 ± 2.01 ^g,h^	4.90 ± 2.09 ^d^	5.35 ± 1.72 ^d,e,f^
FolgersCB	4.13 ± 2.13 ^c,d,e^	5.83 ± 1.76 ^b,c^	5.53 ± 1.49 ^c,d,e^
FolgersCM	3.18 ± 2.01 ^g,h^	6.84 ± 1.72 ^a^	6.00 ± 1.59 ^b^
KenyaCB	3.53 ± 1.87 ^f,g,h^	4.36 ± 1.94 ^e,f^	5.40 ± 1.60 ^d,e,f^
KenyaCM	3.10 ± 1.93 ^h^	5.04 ± 2.14 ^d^	5.82 ± 1.51 ^b,c^
BaristaRTD(A) ^3^	5.54 ± 2.15 ^a^	4.23 ± 1.82 ^e,f^	5.18 ± 1.74 ^e,f^
BaristaRTD(B)	5.80 ± 1.90 ^a^	4.14 ± 1.77 ^e,f^	5.01 ± 1.79 ^f^
BabinskiRTD	5.50 ± 2.04 ^a^	3.89 ± 1.73 ^f^	5.18 ± 1.61 ^e,f^
FrenchCafeRTD	4.65 ± 2.09 ^b,c^	4.07 ± 1.81 ^f^	5.54 ± 1.66 ^c,d,e^
StarbucksCS	4.05 ± 2.12 ^d,e,f^	625 ± 1.82 ^b^	6.01 ± 1.42 ^a,b^
TwosomeplaceCS	4.76 ± 2.47 ^b^	5.69 ± 1.97 ^c^	6.41 ± 1.68 ^a^
*p*-value	<0.0001	<0.0001	<0.0001
LSD	0.52	0.47	0.41

^1^ CB stands for cold brew; CM stands for coffee maker brewed (hot); RTD stands for ready-to-drink; CS stands for coffee shop samples. ^2^ Mean ± SD. ^3^ BaristaRTD(A) and BaristaRTD(B) were evaluated twice as blind duplicate samples for evaluations divided into two sessions to measure consumers’ consistency. Same characters in the same column mean no significant difference by ANOVA at 95% confidence level.

**Table 9:** Correlation matrix between volatile compounds and sensory data, caffeine, chlorogenic acid, and trigonelline compounds ^1^.

No.	Volatile Compounds	Overalllliking	Bitterness Intensity	CoffeeID Intensity	Caffeine	Chlorogenicacid	Trigonelline
V1	Pyridine	0.4051	0.4506	**0.6294**	0.4009	−0.1188	0.0673
V2	Methylpyrazine	−0.2472	**0.8086**	0.4608	**0.8199**	0.5801	**0.6219**
V3	Furfural	**−0.6355**	−0.1263	−0.2608	−0.1285	0.4423	0.3804
V4	2-Furan methanol	−0.0859	**0.7601**	0.5833	**0.7383**	0.4578	0.5857
V5	2,6-Dimethylpyrazine	−0.2948	**0.8019**	0.4662	**0.8076**	**0.6103**	**0.6574**
V6	3-Ethylpyridine	0.4834	0.3109	0.5360	0.2328	−0.3096	−0.1300
V7	5-Methyl-2-furaldehyde	**−0.6712**	0.8121	−0.1314	0.1091	0.5790	0.5186
V8	Phenol	0.1530	**0.6796**	0.7254	**0.6269**	0.1019	0.2635
V9	2-ethyl-3-methylpyrazine	−0.1883	**0.7410**	0.4601	**0.7540**	0.4611	0.5144
V10	Pyrrole-2-carboxaldehyde	−0.3690	0.5661	0.5806	0.5901	0.5247	**0.6039**
V11	1-methyl-1H-pyrrole-2-acetonitrile	−0.4306	**0.6318**	0.2789	**0.6562**	**0.6218**	**0.6298**
V12	N-acetyl-4(H)-pyridine	−0.1002	**0.8564**	0.5421	**0.8420**	0.4702	0.5520
V13	3-Methylphenol	**0.6201**	0.1255	0.3068	0.1737	−0.2674	−0.1545
V14	2-Acetylpyrrole	0.0736	**0.7221**	**0.7068**	**0.6866**	0.2262	0.3815
V15	3,5-Octadien-2-one	**−0.6605**	−0.001	−0.1487	−0.0081	0.5058	0.4606
V16	2-Acetyl-1-methylpyrrole	0.0652	**0.6320**	**0.6768**	0.5811	0.1094	0.2566
V17	3-Ethyl-2,5-dimethylpyrazine	−0.1762	**0.6566**	0.4092	**0.6729**	0.4115	0.4399
V18	2-Ethyl-3,5-dimethylpyrazine	−0.1167	**0.6941**	0.4635	**0.7085**	0.3795	0.4271
V19	2,5-Diethylpyrazine	−0.1665	**0.7169**	0.4614	**0.7351**	0.4239	0.4626
V20	Guaiacol	0.0527	**0.7366**	**0.6933**	**0.6967**	0.1917	0.3222
V21	3-Ethyl-2-hydroxy-2-cyclopenten-1-one	−0.3873	**0.6740**	0.5526	**0.6574**	0.5963	**0.6996**
V22	Nonanal	0.0994	0.5327	0.3874	0.5662	0.1145	0.1230
V23	Maltol	0.0657	0.4424	0.4479	0.4167	0.1943	0.3384
V24	2-Methyl-5-propionylfuran	0.2251	0.4188	**0.6402**	0.4146	0.0515	0.2315
V25	5H-5-methyl-6,7-dihydrocyclopenta[b]pyrazine	−0.1519	**0.7948**	0.5582	**0.7790**	0.4000	0.4689
V26	1-Furfuryl pyrrole	−0.1563	0.5910	0.1064	0.5744	0.2252	0.1986
V27	Methyl salicylate	−0.2344	0.4278	0.1778	0.3649	0.4178	0.4739
V28	Nonanoic acid	−0.0295	**0.7255**	0.4077	**0.6927**	0.2501	0.3134
V29	4-Ethylguaiacol	−0.1073	**0.7424**	0.4100	**0.7242**	0.2966	0.3288
V30	Difurfuryl ether	0.2240	0.5974	0.4923	0.5505	−0.0282	0.0975
V31	4-Vinylguaiacol	**−0.6003**	**0.6164**	0.1443	0.5763	0.5454	0.4771
V32	Decanoic acid	−0.2637	**0.7282**	0.4161	**0.7641**	0.4482	0.4652
V33	3,4-Dimethoxystyrene	−0.5263	0.4180	0.2003	0.4760	0.5291	0.5141
V34	2,4-Di-tert-butylphenol	0.0732	0.5252	0.6752	0.5544	0.1575	0.2564
V35	Dodecanoic acid	−0.5723	0.4563	0.4057	0.4021	0.5898	**0.6323**
V36	Tetradecanoic acid	−0.3523	−0.2690	−0.2988	−0.2671	0.1716	0.1626

^1^ Correlation coefficients (>|0.6|) are indicated in bold.
